# Sclerosing Encapsulating Peritonitis Is More Common Following Repeat Hyperthermic Intraperitoneal Chemotherapy

**DOI:** 10.7759/cureus.105647

**Published:** 2026-03-22

**Authors:** Ariel Davydov, Salvador Rodriguez Franco, Steven Ahrendt

**Affiliations:** 1 Division of Surgical Oncology, Department of Surgery, University of Colorado Anschutz Medical Campus, Aurora, USA

**Keywords:** cytoreductive surgery (crs), hyperthermic intraperitoneal chemotherapy, peritoneal metastasis, repeat cytoreductive surgery, sclerosing encapsulating peritonitis (sep)

## Abstract

Introduction: Sclerosing encapsulating peritonitis (SEP) is a rare complication of cytoreductive surgery (CRS) with hyperthermic intraperitoneal chemotherapy (HIPEC). The purpose of this study is to determine the incidence and risk factors for developing SEP, as well as to characterize the clinical presentation, diagnosis, and management outcomes of SEP.

Methods: This study was conducted at the University of Colorado Anschutz in Aurora, Colorado, a tertiary referral center for peritoneal surface malignancies. Patients undergoing CRS with or without HIPEC were identified from our retrospective CRS/HIPEC database. Patients with clinical, operative, and/or radiologic features of SEP were identified, and their clinical parameters were compared with those of patients without these findings.

Results: Between January 1, 2017, and June 1, 2025, 500 patients underwent CRS with (n = 388) or without HIPEC (n = 112). Six patients developed the typical clinical and radiologic findings of SEP, including chronic or recurring small bowel obstruction, postprandial abdominal pain, vomiting, weight loss, and persistent small bowel dilation on CT scans. The median time from the most recent CRS/HIPEC to presentation with SEP was 5.5 months (range 2-11). Five of six patients underwent laparotomy, with four receiving surgical resection of the encapsulating fibrous rind. Symptoms resolved in four patients with no recurrence. One patient developed a chronic enterocutaneous fistula and is total parenteral nutrition (TPN) dependent. One patient opted for observation and has ongoing symptoms of SEP. SEP was significantly more common (p < 0.05) in patients following repeat (>2) HIPEC (3/53, 5.7%) than an initial HIPEC (3/335, 0.9%).

Conclusions: SEP is a rare complication of HIPEC that presents within a year of HIPEC with chronic or recurrent bowel obstruction secondary to adhesions encasing the small bowel. The risk of SEP increases with repeat CRS/HIPEC.

## Introduction

Sclerosing encapsulating peritonitis (SEP) is a rare complication of cytoreductive surgery (CRS) with hyperthermic intraperitoneal chemotherapy (HIPEC) [1,2]. This phenomenon is believed to be secondary to chronic inflammation that promotes the formation of a fibrous capsule encasing the small bowel, resulting in symptoms of small bowel obstruction. It has been reported with HIPEC using common chemotherapeutic agents [2]. The purpose of this study is to determine the incidence and risk factors for developing SEP. Secondary aims include characterizing the clinical presentation, diagnosis, and management outcomes of SEP.

## Materials and methods

Patients undergoing CRS with or without HIPEC between January 1, 2017, and June 1, 2025, were identified from our prospective CRS/HIPEC database. This study was conducted at the University of Colorado Anschutz in Aurora, Colorado, a tertiary referral center for peritoneal surface malignancies. Inclusion criteria comprised adult patients (≥18 years) who underwent CRS with or without HIPEC for peritoneal surface malignancy. Exclusion criteria included patients with incomplete operative records, follow-up less than 30 days, or preexisting sclerosing peritonitis. The HIPEC protocol was selected based on the patient’s tumor type, with 90% of patients managed with HIPEC receiving 40 mg mitomycin-C for 100 minutes with a goal peritoneal temperature of 41-42°C. Disease burden was quantified using the Peritoneal Cancer Index (PCI), which divides the abdomen into 13 regions and assigns lesion size scores (0-3) in each region, yielding a total score from 0 to 39, with higher scores indicating greater tumor burden [3]. Completeness of cytoreduction (CC) was graded using the CC score: CC-0 (no visible disease), CC-1 (residual nodules ≤2.5 mm), CC-2 (nodules 2.5 mm-2.5 cm), and CC-3 (nodules >2.5 cm) [3]. CC-0/1 was considered a complete cytoreduction.

Patients with clinical, operative, and/or radiologic features of SEP were identified, and their clinical parameters were compared with those of patients without these findings. Six patients developed the typical clinical and radiologic findings of SEP, including chronic or recurrent small bowel obstruction, postprandial abdominal pain, nausea/vomiting, weight loss, and persistent small bowel dilation on CT scans. The electronic medical records of these six patients were reviewed for baseline characteristics, operative details of CRS/HIPEC, presentation, radiologic findings, management, and follow-up. Data collection and analysis were conducted with approval from the Colorado Multiple Institutional Review Board (COMIRB #18-2839 and #22-1446).

Categorical variables were analyzed using the chi-square test or Fisher's exact test. A two-sided p-value < 0.05 was considered statistically significant. Effect sizes were calculated as risk differences and risk ratios with exact confidence intervals; a continuity correction was applied for zero-event comparisons. Statistical analyses were performed using R version 4.5.2 (R Foundation for Statistical Computing, Vienna, Austria).

## Results

Between January 1, 2017, and June 1, 2025, 500 patients underwent CRS with (n = 388) or without HIPEC (n = 112). Of these patients, six presented with clinical, radiologic, and operative findings of SEP. The clinical characteristics of these six patients are detailed in Table 1. All six patients with SEP received HIPEC with mitomycin-C prior to presentation. The median time from the most recent CRS/HIPEC to presentation with SEP was 5.5 months (range 2-11). Patients initially presented with symptoms of persistent or recurring small bowel obstruction: significant postprandial abdominal pain, lack of appetite, nausea/vomiting, and weight loss.

**Table 1:** Clinical characteristics of patients presenting with SEP following CRS/HIPEC. LAMN, low-grade appendiceal mucinous neoplasm; G2, grade 2; CRS, cytoreductive surgery; HIPEC, hyperthermic intraperitoneal chemotherapy; PCI, peritoneal cancer index; CC, completeness of cytoreduction; SBO, small bowel obstruction; Recurrent SBO, episodic pain, vomiting, CT evidence of SBO with complete resolution of symptoms; Chronic SBO, persistent pain, vomiting, weight loss, and CT evidence of SBO without resolution of symptoms; OSH, outside hospital; SEP, sclerosing encapsulating peritonitis; NED, no evidence of disease; AWD, alive with disease

	Patient 1	Patient 2	Patient 3	Patient 4	Patient 5	Patient 6
Diagnosis	Colon adenocarcinoma	LAMN	Appendiceal mucinous (G2) adenocarcinoma	Colon adenocarcinoma	Appendiceal mucinous (G2) adenocarcinoma	LAMN
Age at first CRS/HIPEC	52	58	55	57	82	67
Sex	M	F	M	M	M	F
Agent for initial HIPEC	Mitomycin-C	Mitomycin-C	Mitomycin-C	Mitomycin-C	Mitomycin-C	Mitomycin-C
PCI for initial HIPEC	14	26	23	3	25	36
CC score for initial HIPEC	0	0	0	0	0	1
Interval to repeat HIPEC (months)	n/a	40	19	68	n/a	n/a
Agent for repeat HIPEC	n/a	Mitomycin-C	Mitomycin-C	Mitomycin-C	n/a	n/a
Number of HIPECs	1	2	2	2	1	1
PCI for repeat HIPEC	n/a	4	25	3	n/a	n/a
CC score for repeat HIPEC	n/a	0	1	0	n/a	n/a
Presentation	Chronic SBO	Recurrent SBO	Chronic SBO	Chronic SBO	Chronic SBO	Recurrent SBO
Interval to presentation (months from CRS/HIPEC)	6	11	2	3	7	5
Treatment	Surgery	Surgery	Surgery	Aborted surgery	Surgery	Aborted surgery at OSH
Interval to SEP surgery (months from CRS/HIPEC)	10	13	8	6	9	5
Recurrence of symptoms	No	No	No	No	No	Yes
Follow-up since last CRS/HIPEC (months)	66	18	38	51	34	33
Current survival status	NED	NED	NED	NED	AWD, lung metastases, no peritoneal recurrence	NED
Current fistula	No	Yes	No	No	No	No
Current ostomy	No	Yes	No	No	No	No

While presentation and obstructive findings on imaging raised suspicion for SEP, diagnosis was confirmed by exploratory laparotomy in five of six patients at our institution. Each of these five patients underwent laparotomy, with four receiving lysis of adhesions and resection of the encapsulating fibrous rind. Intraoperative findings revealed dense fibrous adhesions encasing a portion of the small bowel. The goal of surgery was to remove as much of the fibrous rind as technically feasible. Often this required repair of multiple partial and full-thickness enterotomies. Operative treatment was aborted in one of the five patients explored at our institution, whose small bowel adhesions could not be safely dissected. Of the four patients who completed operative treatment, one underwent a second reoperation for an enteric leak that was managed with small bowel resection and diverting enterostomy. One patient was explored for small bowel obstruction at an outside hospital, and the procedure was aborted due to a frozen abdomen. She has since been managed nonoperatively with ongoing intermittent episodes of small bowel obstruction and persistent weight loss. Pathologic evaluation of excisions demonstrated dense fibrocollagenous tissue, consistent with adhesions.

Symptoms resolved in four patients with weight gain, resolution of obstructive symptoms, and no recurrence. One patient developed a chronic enterocutaneous fistula and is total parenteral nutrition (TPN) dependent. One patient opted for observation and has ongoing symptoms of SEP. 

The incidence of SEP was 1.5% following CRS/HIPEC compared with 0% after CRS alone (risk difference 1.5%, 95% CI -0.2-3.3; RR 3.8, 95% CI 0.2-66) (Table 2). SEP occurred in 1.7% of mitomycin-C cases versus 0% of platinum-based HIPEC (risk difference 1.7%, 95% CI -0.1-3.6; RR 2.8, 95% CI 0.2-48). Repeat CRS/HIPEC demonstrated the highest risk, with SEP in 5.7% compared with 0.9% after the first CRS/HIPEC (risk difference 4.8%, 95% CI 0.2-9.4; RR 6.3, 95% CI 1.3-30). SEP was significantly more common (p < 0.05) in patients undergoing their second or greater HIPEC.

**Table 2:** Incidence of SEP development by CRS/HIPEC status and regimen. Statistical comparisons were performed using chi-square or Fisher's exact tests for categorical variables. Effect sizes were calculated as risk differences and risk ratios with exact confidence intervals; a continuity correction was applied for zero-event comparisons. SEP, sclerosing encapsulating peritonitis; CRS, cytoreductive surgery; HIPEC, hyperthermic intraperitoneal chemotherapy (*: p ≤ 0.05, **: p ≤ 0.01, ***: p ≤ 0.001)

	No. of patients developing SEP (%)	Risk difference (95% CI)	Risk ratio (95% CI)	Chi-square value	Fisher's exact test
CRS/HIPEC	6/388 (1.5%)	+1.5% (−0.2-3.3)	3.8 (0.2-66)	0.35	–
CRS w/o HIPEC	0/112 (0%)				
Mitomycin-C HIPEC	6/349 (1.7%)	+1.7% (−0.1-3.6)	2.8 (0.2-48)	1.0	–
Oxaliplatin or Cisplatin HIPEC	0/39 (0%)				
First CRS/HIPEC	3/335 (0.9%)	+4.8 (0.2-9.4)	6.3 (1.3-30)	–	0.04*
Second or higher CRS/HIPEC	3/53 (5.7%)				

## Discussion

SEP is a rare (1-2%) complication of HIPEC that presents within a year of HIPEC with chronic or recurrent bowel obstruction secondary to adhesions encasing the small bowel. It was first defined in 1907 by Owtschinnikow as “peritonitis chronica fibrosis incapsulata” and has additionally been referred to as “abdominal cocoon” and “abdominal cocoon syndrome” [4,5]. While the pathogenesis of SEP has remained relatively unknown, it is largely believed to be secondary to chronic inflammation that promotes the formation of a fibrous capsule encasing the small bowel [1,2]. SEP has been divided into primary and secondary forms according to the underlying etiology [4]. Primary SEP is idiopathic and characteristically seen in young adolescent females from tropical and subtropical countries, while secondary SEP has most commonly been recognized as a severe complication of continuous ambulatory peritoneal dialysis (CAPD), affecting between 1.4% and 7.3% of patients undergoing CAPD [5-7]. Other reported factors associated with SEP include prior abdominal surgery, bacterial peritonitis, organ transplantation, and chronic use of beta-blockers [8,9].

Secondary SEP has also been associated with intraperitoneal chemotherapy (IPC) and HIPEC in rare case reports of patients receiving HIPEC for metastatic colon, appendiceal, gastric, and ovarian cancer, as well as peritoneal mesothelioma [8]. This was first reported by Markman et al., who suggested increased risk of peritoneal sclerosis and extensive adhesion formation with the use of IPC [10]. To our knowledge, 14 cases of SEP have been reported in association with HIPEC, as of 2025 [1,2,8,9,11]. SEP has been reported with HIPEC using common chemotherapeutic agents, including mitomycin-C, oxaliplatin, and cisplatin [8].

The cause of secondary SEP in patients treated with CRS and HIPEC is unclear, although Liberale and Sugarbaker hypothesized several possible etiologies in a 2018 case series: CRS with extensive peritonectomy, the hyperthermic nature of the infusion, chemotherapy agents used during HIPEC, or a combination of these factors [2]. While our data did not demonstrate a significant difference in the incidence of SEP in CRS with or without HIPEC, the incidence of SEP in our cohort significantly increased with repeat HIPEC, suggesting that increased exposure to intraperitoneal chemotherapeutic agents or hyperthermic infusion can contribute to the risk of acquiring SEP.

Increased risk of peritoneal fibrosis with numerous cycles of IPC was described by Jaquet et al. in a 1995 retrospective study that reviewed 196 patients who received at least one cycle of early postoperative IPC with 5-fluorouracil (5-FU) and IPC mitomycin-C [12]. Within this cohort, 152 patients received three additional cycles of IPC 5-FU and systemic mitomycin-C, while 35 received three additional cycles of IPC 5-FU and IPC mitomycin-C [12]. Of the patients who received 4 cycles of IPC mitomycin-C, three patients (9%) developed peritoneal fibrosis that ultimately required reoperation due to chronic bowel obstruction secondary to dense adhesions. Only one patient (0.01%) of the 152 who received three additional cycles of IPC 5-FU and systemic mitomycin-C developed adhesions requiring reoperation. Jaquet et al. concluded that numerous cycles of IPC mitomycin-C may predispose patients to developing a “long-term adhesive process in the abdominal cavity” (now understood as SEP) and suggested that repeat cycles of mitomycin-C should be avoided as a result [12].

While Jaquet et al. evaluated the impact of numerous cycles of IPC on the risk of peritoneal fibrosis or SEP, a limitation of their study is that outcomes with repeat cycles of IPC were not compared to those of patients undergoing one cycle of IPC. Though it has been shown that there is an increased risk of SEP with numerous cycles of IPC versus systemic chemotherapy, there has yet to be a study evaluating the specific contribution of additional cycles of HIPEC on the risk of SEP in patients who have previously received CRS with HIPEC. Our data demonstrates a significant six-fold increase in risk of SEP in patients who received two or more courses of HIPEC (5.7%) when compared to those who received one course of HIPEC (0.9%). We found no statistically significant difference in our cohort in the incidence of SEP when comparing different chemotherapeutic agents, specifically mitomycin-C, cisplatin, or oxaliplatin (Table 2).

Clinically, SEP commonly presents as recurrent acute, subacute, or chronic small bowel obstruction with symptoms of abdominal pain. Patients with SEP present relatively quickly following HIPEC, as all six of our patients presented within a year of their initial operation. It is important to note that no specific diagnostic criteria exist for SEP [2]. However, the gold standard for diagnosis is laparotomy with classic findings of dense fibrotic adhesions and capsules encasing the bowel and confirmatory examination on pathology demonstrating fibrous tissue [2,13]. Preoperative diagnosis is often difficult, as symptoms and radiologic findings may be non-specific [13,14]. As current diagnostic criteria for SEP rely heavily on clinical judgment rather than standardized definitions, further studies establishing objective diagnostic criteria for SEP are indicated. The other diagnostic consideration is progression of the patient’s peritoneal malignancy, as worsening abdominal pain and weight loss are also present in this clinical scenario. In both SEP and early cancer progression, clear evidence of peritoneal metastases is absent on CT imaging. In several patients in this series, the clinical picture of disease progression and malignant bowel obstruction was at odds with the expected excellent clinical outcome (low-grade disease, low PCI) following their CRS/HIPEC. Abdominal X-ray and CT imaging in patients with SEP commonly demonstrate findings suggestive of bowel obstruction, including dilated small bowel with air fluid levels [15]. In our experience, patients with SEP develop a fixed, consistent pattern of small bowel dilation that persists across serial CT scans. Several patients in this series had proximal small bowel involvement with relative sparing of the distal small bowel. In two patients explored at five and six months following CRS/HIPEC, dense adhesions precluded a safe procedure, and the laparotomy was aborted. The operative procedure was more likely to be successful when completed between eight and 13 months after CRS/HIPEC.

SEP is classically treated with surgical removal of the fibrous capsule, although this operation may be challenging. The capsule can be densely adherent, and partial and full-thickness enterotomies are common. In several of the patients in this series, dramatic clinical improvement was noted with resolution of all symptoms despite partial removal of the capsule and numerous enterotomy repairs. Nevertheless, a common adverse event associated with surgical resection of SEP is small bowel fistula [2]. Surgical management was aborted in one of our five patients to avoid this outcome, as his small bowel could not be safely dissected. His symptoms resolved without further operative intervention. The four patients who completed surgical management of SEP resolved postoperatively.

This study should be interpreted in the context of several limitations. Our retrospective single-center design introduces potential selection bias, precludes adjustment for potential confounders, and limits generalizability. Unmeasured confounders such as variations in operative technique, choice of HIPEC regimen, and postoperative management may have influenced outcomes. Additionally, the small number of SEP events resulted in limited statistical power and widened confidence intervals, which increases the risk of type II error. Strengths of the study include a relatively large cohort of 500 patients, as well as being the first of its kind to assess incidence and risk factors for SEP.

## Conclusions

SEP is a rare complication associated with HIPEC with increased risk following repeat CRS/HIPEC. There is no difference in the risk of SEP among mitomycin-C, oxaliplatin, and cisplatin-based HIPEC. Consistent with prior reports, surgical management can be curative; however, the risk of enterocutaneous fistula is high. The natural history of patients with SEP undergoing nonoperative management remains unclear. Future studies are needed to establish objective diagnostic thresholds and define nonoperative management strategies.
